# Metabolic bifunctionality of Rv0812 couples folate and peptidoglycan biosynthesis in *Mycobacterium tuberculosis*

**DOI:** 10.1084/jem.20191957

**Published:** 2021-05-05

**Authors:** Katherine A. Black, Lijun Duan, Lungelo Mandyoli, Bruna P. Selbach, Weizhen Xu, Sabine Ehrt, James C. Sacchettini, Kyu Y. Rhee

**Affiliations:** 1 Weill Cornell Medicine, New York, NY; 2 Texas A&M University, College Station, TX

## Abstract

Comparative sequence analysis has enabled the annotation of millions of genes from organisms across the evolutionary tree. However, this approach has inherently biased the annotation of phylogenetically ubiquitous, rather than species-specific, functions. The ecologically unusual pathogen *Mycobacterium tuberculosis* (*Mtb*) has evolved in humans as its sole reservoir and emerged as the leading bacterial cause of death worldwide. However, the physiological factors that define *Mtb’*s pathogenicity are poorly understood. Here, we report the structure and function of a protein that is required for optimal in vitro fitness and bears homology to two distinct enzymes, Rv0812. Despite diversification of related orthologues into biochemically distinct enzyme families, *rv0812* encodes a single active site with aminodeoxychorismate lyase and D–amino acid transaminase activities. The mutual exclusivity of substrate occupancy in this active site mediates coupling between nucleic acid and cell wall biosynthesis, prioritizing PABA over D-Ala/D-Glu biosynthesis. This bifunctionality reveals a novel, enzymatically encoded fail-safe mechanism that may help *Mtb* and other bacteria couple replication and division.

## Teaser

Despite diversification into biochemically distinct enzyme families, rv0812 encodes a single active site that functions as an enzymatically bifunctional aminodeoxychorismate lyase (ADCL) and D–amino acid transaminase (DAAT) and may couple replication and division in *Mycobacterium tuberculosis*.

## Introduction

Despite Koch’s discovery of *Mycobacterium tuberculosis* (*Mtb*) as the causative agent of tuberculosis (TB) over 130 yr ago, TB remains the leading cause of death due to infectious disease and has emerged as the leading cause of death due to antibiotic resistance (World Health Organization, 2020). Surprisingly, knowledge of *Mtb*-specific physiology remains incomplete. High-throughput sequencing technologies have helped to overcome this barrier (Cole et al., 1998; Saghatelian and Cravatt, 2005); however, it is estimated that approximately one third to one half of all *Mtb* genes lack a functional annotation, are potentially misannotated, or encode noncanonical activities beyond their predicted functions (Hanson et al., 2009).

Current bioinformatic sequence annotations, to a great extent, are based on sequence homology. These methods derive their utility from phylogenetically conserved structure–function relationships. While powerful, these methods are associated with an underappreciated bias favoring the annotation of evolutionarily invariant functions, leaving the most specific features of a given organism’s physiology undefined (Cole et al., 1998; Lechartier et al., 2014). This bias is amplified further by its extension to genes exhibiting intermediate degrees of sequence conservation, where annotations of ancestral over species-specific functions are favored, and when functions are identified, they are often restricted to a general class level.

Here, we investigated the function of *rv0812*, a gene that was predicted to be essential for in vitro growth of *Mtb* and found to be upregulated within the lungs of infected mice, but that bears homology to two biochemically distinct enzymes (DeJesus and Ioerger, 2013; DeJesus et al., 2017b; Dubnau et al., 2005; Griffin et al., 2011; Zhang et al., 2012). Bioinformatic sequence analysis identified Rv0812 as a member of the type IV family of pyridoxyl-5′-phosphate (PLP)–dependent enzymes (PLPDEs), with nearly equivalent degrees of homology to bacterial aminotransferases involved in synthesis of cell wall–associated D–amino acids and enzymes involved in the synthesis of the folate precursor para-aminobenzoic acid (PABA; Cole et al., 1998; Kapopoulou et al., 2011; Lew et al., 2011; Pruitt et al., 2007; Wattam et al., 2014). Previous work implicated a role for Rv0812 in the latter (Chim et al., 2011; Thiede et al., 2016). Using a combination of biochemistry, metabolomics, structural biology, and chemical genetics, we demonstrate that Rv0812 catalyzes two enzymatic reactions in biochemically unrelated pathways. One is as an aminodeoxychorismate (ADC) lyase (ADCL) involved in folate biosynthesis. The other is as a D–amino acid transaminase (DAAT) involved in peptidoglycan (PG) biosynthesis. All reported ADCLs and DAATs have been found to be encoded by separate genes (Jhee et al., 2000; Mehta and Christen, 2000; Miles, 1985). The bifunctionality of Rv0812 suggests that *Mtb* has encoded one enzyme with both activities to mechanistically buffer against the competing metabolic demands of nucleic acid and cell wall biosynthesis in a manner that may ensure the orderly progression of *Mtb* replication and division.

## Results and discussion

### Biochemical characterization of recombinant Rv0812

Comparative sequence analysis annotates Rv0812 as a type IV PLPDE (Cole et al., 1998). PLP, the biologically active form of vitamin B6, is an enzymatic cofactor used by one of the most diverse superfamilies of enzymes and is involved in the catalysis of a wide range of transamination, decarboxylation, deamination, and racemization reactions (Christen and Mehta, 2001). The type IV family of PLPDEs is distinguished by a stereochemically conserved active site architecture that promotes *re*-face rather than *si*-face proton transfer relative to the C4′ of the planar pi system of the cofactor (Jhee et al., 2000; Martínez del Pozo et al., 1989a; Sugio et al., 1995; Yoshimura et al., 1993). Annotated members of the type IV family include ADCL, DAAT, branched chain amino acid transaminase, and R-stereospecific amine transaminase (Percudani and Peracchi, 2009), each of which appears to have divergently evolved distinct substrate and reaction specificities.

**Figure 1.**
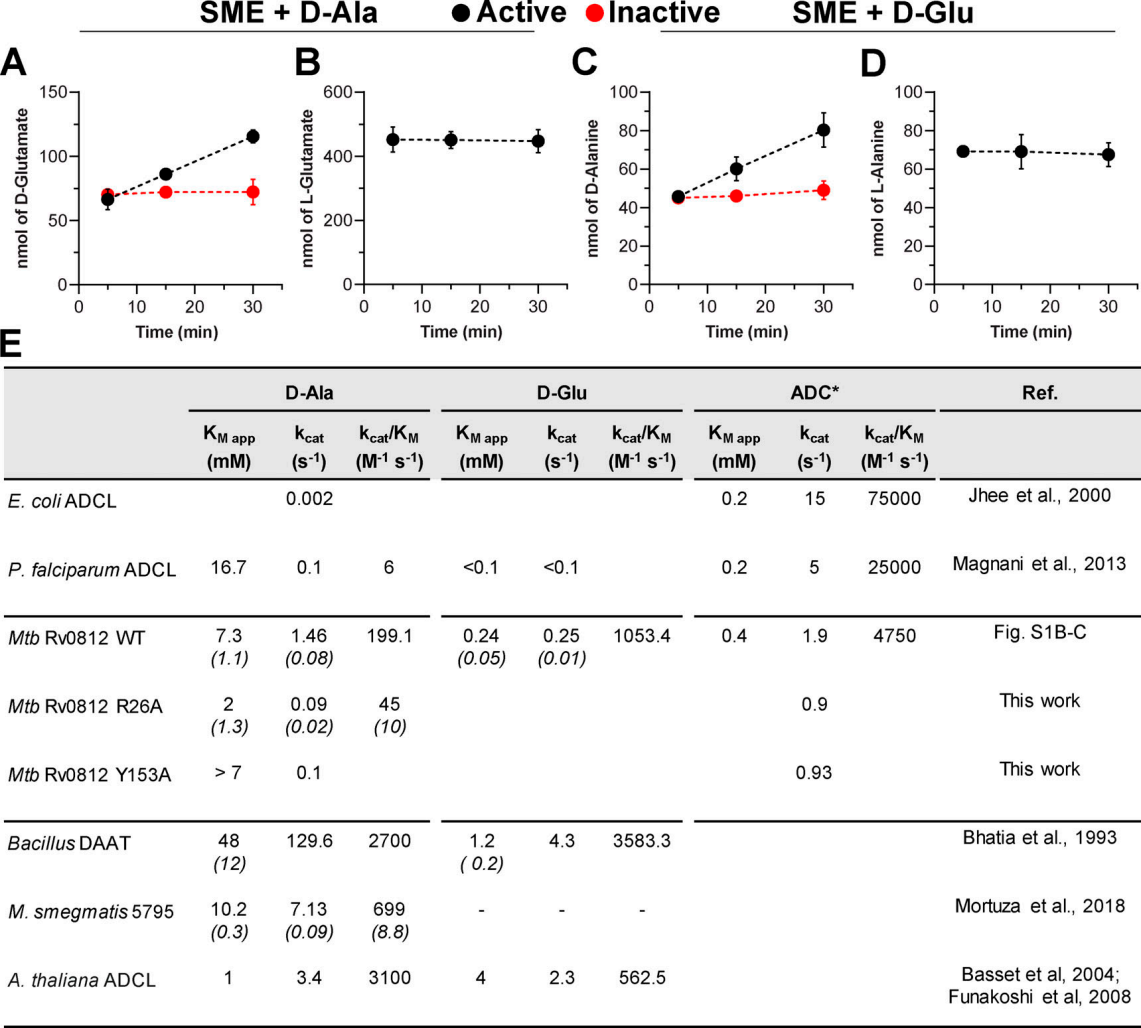
**Rv0812 is a bifunctional enzyme with DAAT and ADCL activities.** ABMP experiments revealed the DAAT activity of Rv0812. **(A–D)** Reactions (150 µl) containing 75 µl SME substrate, 50 µM PLP, 1 mM D-Ala (A and B) or D-Glu (C and D), and 5 µM Rv0812 were monitored by quenching aliquots at various time points for LC-MS analysis. Accumulation of D-Ala or D-Glu was dependent on the presence of active enzyme and had no impact on the concentration of L-enantiomers present in the SME. **(E)** Kinetic parameters for *Mtb* Rv0812 WT and variants were determined with pure substrates and compared with those of known DAAT and ADCL enzymes. SDs are shown inside parentheses in italics. (Kinetic parameters with ADC as substrate are reported as rough approximations, as ADC instability prevents well-defined substrate concentrations and, thus, accurate kinetic determination.) SDs were obtained from at least three independent experiments.

In contrast to most type IV PLPDEs, Rv0812 exhibits equivalent degrees of sequence homology to both ADCLs and DAATs. Published work has implicated Rv0812 in each role (Chim et al., 2011; DeJesus et al., 2017a; Kieser et al., 2015; Thiede et al., 2016; Xu et al., 2017). We sought to resolve this ambiguity by characterizing the in vitro activity of purified recombinant Rv0812. Consistent with previous phenotypic studies of a Rv0812 transposon mutant and biochemical assays of a purified preparation of recombinant Rv0812, we first confirmed Rv0812’s enzymatic activity as an ADCL (Chim et al., 2011). Owing to the intrinsic chemical instability of the substrate ADC, which precluded determination of absolute kinetic parameters, we assayed for ADCL activity using a published coupled assay in which ADC was generated in situ through the addition of chorismate to purified *Escherichia coli* ADC synthase (*pabB*), and ADCL activity was detected by spectrophotometric monitoring of pyruvate production using lactate dehydrogenase (LDH; Ye et al., 1990). We observed activity levels similar to those reported for the *E. coli* and *Plasmodium falciparum* ADCL enzymes, with Rv0812 catalyzing ADC turnover at 1.9 s^−1^ compared with 15 and 5 s^−1^ for the orthologues from *E. coli* and *P. falciparum*, respectively (Fig. 1 E; Jhee et al., 2000; Magnani et al., 2013).

**Figure S1.**
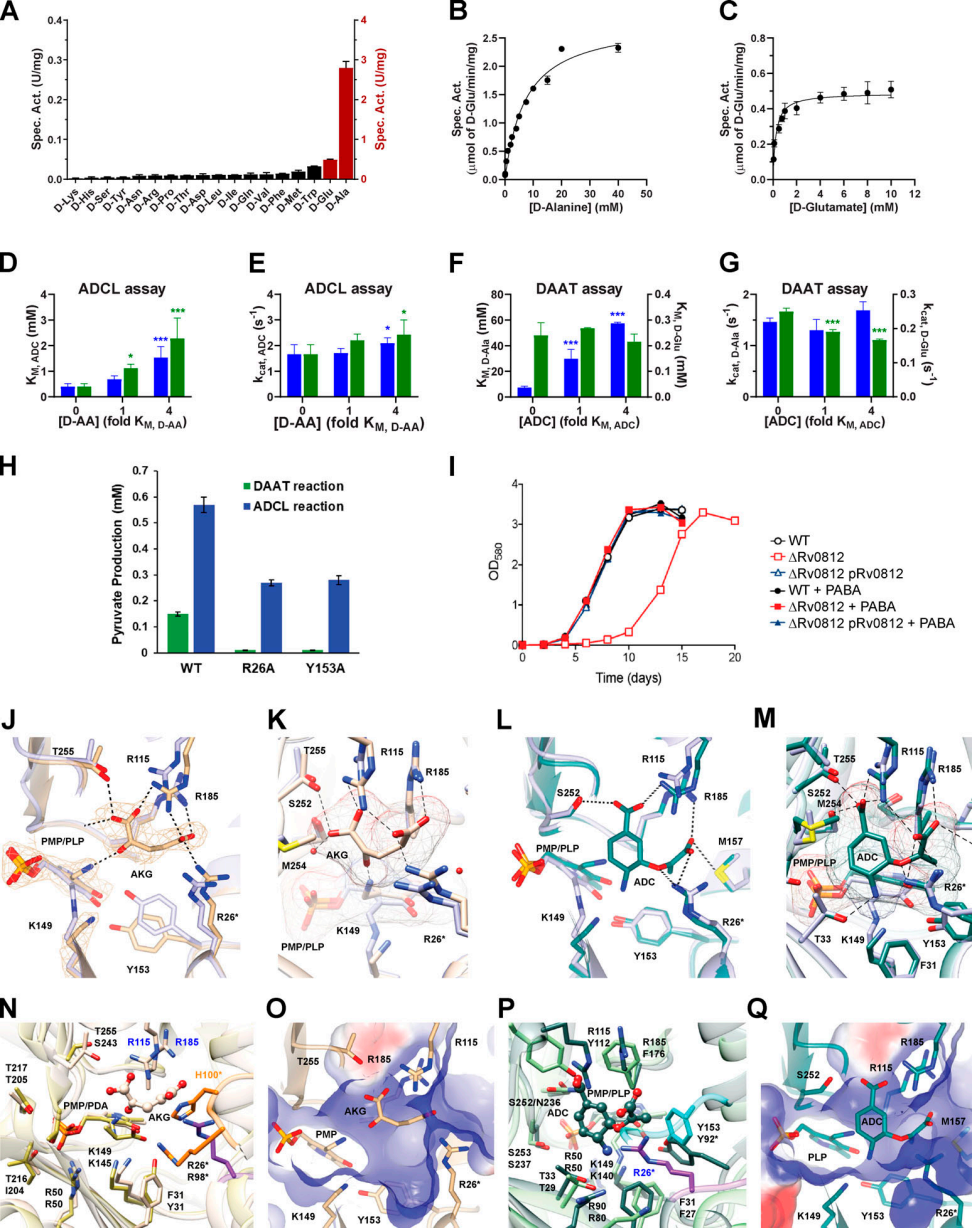
**Structural, kinetic, and phenotypic analysis demonstrate the ability of Rv0812 to serve as both a DAAT and ADCL.** **(A)** Rv0812 DAAT activity is restricted to D-Ala and D-Glu. Reaction aliquots containing 0.5 µM Rv0812, 50 µM PLP, 10 mM AKG, and 1 mM of each D–amino acid were quenched at various time points for LC-MS analysis. Activity was quantified by assessing substrate depletion over time and normalizing to a standard curve. Specific activities with D-Ala and D-Glu are plotted on the right y-axis, and specific activity (spec. act.) with other substrates is plotted on the left y-axis. **(B and C)** Activity profile of Rv0812 DAAT reactions. The rate of pyruvate formation (B) or D-Glu consumption (C) was determined in reactions with 0.25 µM Rv0812, 5 mM AKG or pyruvate, 50 µM PLP, and varied substrate concentrations at pH 8.5. Data were fitted to the Michaelis-Menten equation to give kinetic parameters: (A) V_max_ = 2.8 ± 0.16 U/mg and *K*_M_ = 7.3 ± 1.06 mM; and (B) V_max_ = 0.49 ± 0.019 U/mg and *K*_M_ = 0.24 ± 0.05 mM. **(D–G)** Substrate competition assays demonstrated the impact of DAAT substrates on ADCL activity (D and E) and vice versa (F and G). The x-axis indicates the concentration of D–amino acid or chorismate relative to the *K*_M_. The y-axis indicates the kinetic constants obtained in the presence or absence of competing substrate. Blue and green bars indicate the presence of D-Ala or D-Glu, respectively. Each column represents the mean ± SD (*n* = 6; *, P < 0.05; ***, P < 0.001) compared with parameters in the absence of competing substrate. **(H)** Pyruvate produced in DAAT (green) and ADCL (blue) reactions. **(I)** Growth of WT *Mtb*, *ΔRv0812*, and *ΔRv0812::Rv0812* in the presence or absence of PABA (1 µg/ml). **(J–Q)** Active site of AKG-bound Rv0812 and ADC-docked model. **(J–M)** Superposition of PLP-bound Rv0812 (purple) with AKG-bound form (ivory; J and K), and with ADC-docked model (teal; L and M). The AKG molecule and waters were removed before docking ADC into the active site. The mesh surface of AKG, ADC, and PMP is shown. H-bonds formed with protein side chains are shown as dashed lines (J–M), and the |(2Fo)-(Fc)| electron density map, contoured at 1.2σ, of PLP and AKG (J) is shown as chicken wire. Side chains of residues neighboring AKG and ADC (within 5 Å) are shown (K and M). **(N and P)** Superposition of PDA-containing *Bs*DAAT (yellow) with AKG-bound Rv0812 (N), and superposition of PLP-containing *Pa*ADCL (green) with ADC-docked Rv0812 (coral; P). *Bs*DAAT and *Pa*ADCL interdomain loops are shown in orange and cyan, respectively. The loop from Rv0812 containing Arg26* is shown in purple. Conserved residues forming the active site are labeled in the order of Rv0812/*Bs*DAAT or Rv0812/*Pa*ADCL. **(O and Q)** Electrostatic surface of Rv0812 in AKG-bound form (O) and ADC-docked model (Q).

Multiple transposon mutagenesis-based screens have separately identified Rv0812 in focused studies of *Mtb* peptidoglycan metabolism (DeJesus et al., 2017a; Kieser et al., 2015; Xu et al., 2017). In addition, a recent study in *Mycobacterium smegmatis* discovered a shunt from D-Glu to D-Ala in an *alr* insertion mutant, prompting the proposal of an alternative transaminase (Marshall et al., 2017). Further studies revealed that the Rv0812 orthologue, *MSMEG_5795*, encoded a DAAT activity capable of restoring growth to strains deleted in either glutamate racemase (Δ*murI)* or alanine racemase (Δ*alr*) when overexpressed (Mortuza et al., 2018). We therefore directly assayed *Mtb* Rv0812 for in vitro activity as a DAAT and discovered a time- and enzyme-dependent stereospecific turnover of D-Ala and D-Glu that could also be observed in the presence of a mycobacterial small-molecule extract containing hundreds of potentially competing substrates (Fig. 1, A and D; de Carvalho et al., 2010). Kinetic studies revealed that Rv0812’s DAAT activity is strictly restricted to D-Ala and D-Glu, as a substrate mix containing α-ketoglutarate (AKG) and a combination of all other D–amino acids showed minimal D-Glu production over time (Fig. S1 A).

To determine the catalytic efficiency of this activity, we determined steady-state kinetic parameters for D-Ala (*K*_M_ [Michaelis constant] and *k*_cat_ [turnover number] 7.3 ± 1.1 mM and 1.46 s^−1^, respectively; Fig. S1 B) and D-Glu (*K*_M_ and *k*_cat_ 0.24 ± 0.05 mM and 0.252 s^−1^, respectively; Fig. S1 C). These values are comparable to those reported for the DAAT from the *Bacillus sp.* YM-1 strain (*K*_M_ and *k*_cat_ for D-Ala: 48 ± 12 mM and 129.6 s^−1^; *K*_M_ and *k*_cat_ for D-Glu: 1.2 ± 0.2 mM and 4.3 s^−1^, respectively), which exhibits weaker substrate affinities but higher turnover rates (Fig. 1 E; Bhatia et al., 1993). The *M. smegmatis* homologue of Rv0812, MSMEG_5795 (70% identity), similarly exhibited a comparable kinetic profile (Fig. 1 E; Mortuza et al., 2018). In contrast, a comparison of specificity constants (*k*_cat_/*K*_M_) revealed a preference of Rv0812 for D-Glu over D-Ala (1 vs. 0.2) while the *Bacillus* DAAT exhibited similar values for both substrates (2.7 vs. 3.6).

Substrate competition experiments were conducted to further elucidate the physiological substrate preferences of Rv0812 under conditions in which both ADCL and DAAT substrates were present. This analysis demonstrated that the catalytic efficiency of Rv0812’s ADCL activity was reduced by ∼50% in the presence of saturating concentrations of D-Ala or D-Glu (Fig. S1, D and E). Both substrates mediated this inhibition in a competitive manner, as their presence increased *K*_M_ for ADC (Fig. S1 D) without affecting the turnover rate (Fig. S1 E). Consistent with the higher binding affinity of Rv0812 for D-Glu over D-Ala, the presence of D-Glu imparted a more dramatic increase in Rv0812’s *K*_M_ for ADC (Fig. S1 D). This is further supported by prior reports of D-Glu–mediated ADCL inhibition in the absence of keto-acids (Magnani et al., 2013). Interestingly, in the reverse competition experiments, we found that while ADC similarly acted as a competitive inhibitor of Rv0812’s DAAT activity with D-Ala as a substrate, its presence had little impact on Rv0812’s kinetic parameters for reactions with D-Glu as the substrate (Fig. S1, F and G).

### Structural characterization of Rv0812

**Figure 2.**
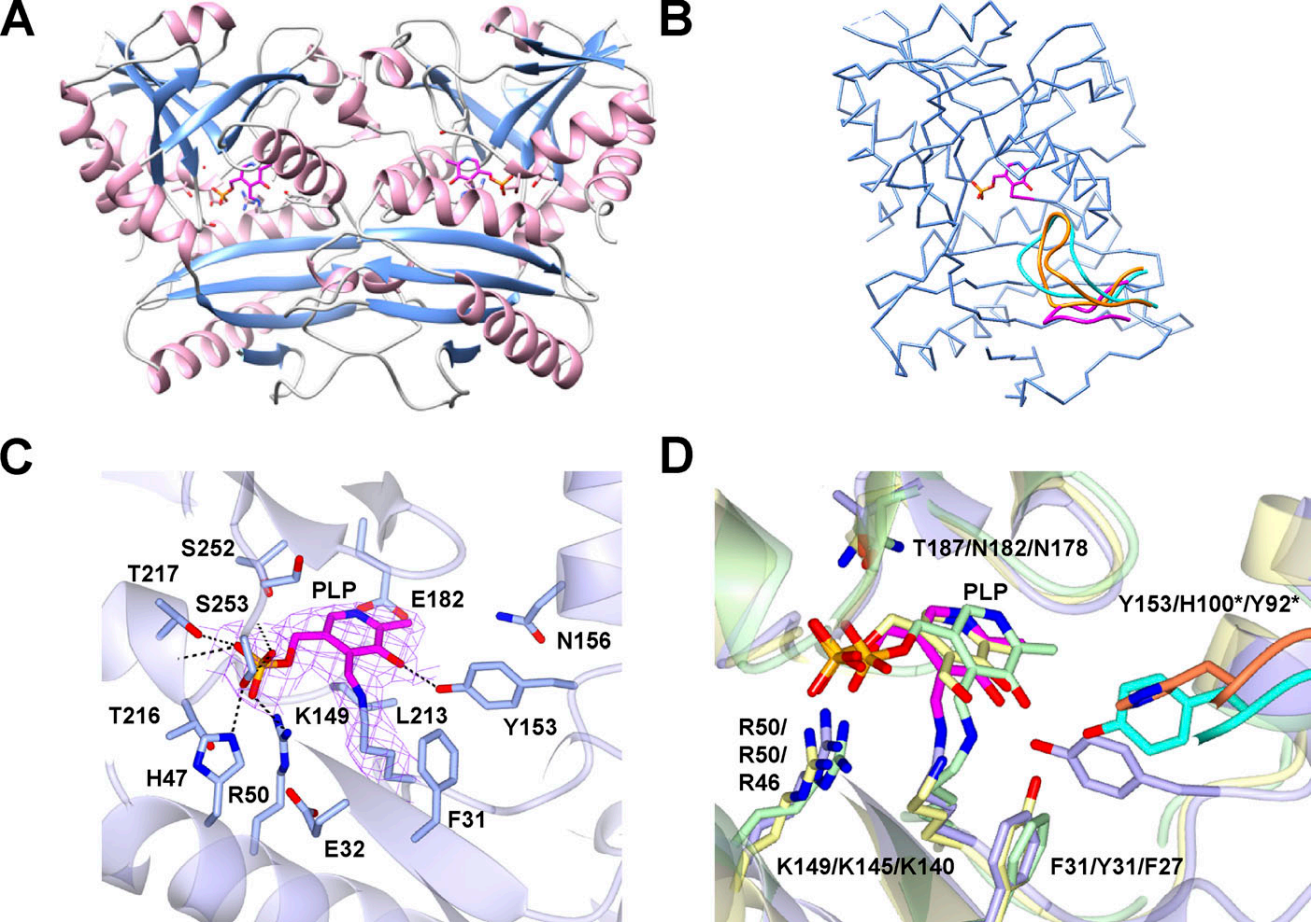
**Structural characterization of Rv0812 reveals the basis for dual ADCL and DAAT activities.** **(A)** Ribbon diagram of PLP-bound dimer (loops in gray, α-helices in pink, and β-sheets in light blue; panels A–D display the PLP molecule in magenta). **(C)** Side chains of PLP-neighboring residues within 5 Å are shown. **(B and** **D)** Structural comparison to *Bs*DAAT and *Pa*ADCL. A shortened loop in Rv0812 (magenta) prevents interaction with the partner subunit active site, as in *Bs*DAAT (orange) and *Pa*ADCL (cyan). **(D)** Active site comparison of PLP-Rv0812 (purple), PLP-bound *Bs*DAAT (yellow), and *Pa*ADCL (green), with residues labeled as Rv0812/*Bs*DAAT/*Pa*ADCL. H-bonds formed between Rv0812 and PLP (C) are shown with dashed lines, and the |(2Fo)-(Fc)| electron density map, contoured at 1.2σ, of PLP and Lys149 (C) is shown as chicken wire.

To elucidate the structural basis of Rv0812’s enzymatic bifunctionality, we determined the x-ray crystal structure of recombinant Rv0812 at 2.4-Å resolution. Crystals of apo-Rv0812 belonged to the primitive monoclinic space group P2_1_ with two molecules in the asymmetric unit, consistent with the behavior of Rv0812 on size exclusion chromatography and of other ADCLs and DAATs that are functional as homodimers in solution (Table S1; Martínez del Pozo et al., 1989b; Padmanabhan et al., 2009; Ye et al., 1990). Crystals of PLP-bound Rv0812 were similarly found to belong to the P2_1_ space group, with four molecules—composed of two homodimers, AB and CD—in the asymmetric unit (Table S1). Molecular replacement using the type IV PLPDE CpuTA1 from *Curtobacterium pusillum* (Protein Data Bank [PDB] accession no. 5K3W) was used to solve and refine the structure of apo-Rv0812. The refined apo-structure was used to solve the structure of Rv0812 with PLP. There was clear electron density extending from the sidechain of Lys149 (Fig. 2 C), predicted to form the Schiff base with PLP. PLP was fit into the corresponding electron density and it was refined with good stereochemistry for all four molecules found in the asymmetric unit (Fig. 2 C). Residues common to type IV PLPDEs and involved in PLP binding, including His47, Arg50, Tyr153, Glu182, and Ser253, were similarly observed (Fig. 2 C; Marchler-Bauer et al., 2017), although residues neighboring the active site differed from those in other PLP-dependent enzymes (Fig. 2 D).

Superimposing the PLP-bound structure of Rv0812 onto representative PLP-bound DAAT and ADCL structures, we identified structural features that distinguished the Rv0812 active site from that of the DAAT from the *Bacillus sp.* YM-1 strain (*Bs*DAAT; Peisach et al., 1998) and ADCL from *Pseudomonas aeruginosa* (*Pa*ADCL, PDB: 2Y4R; O’Rourke et al., 2011). *Bs*DAAT and *Pa*ADCL share 23% and 27% sequence identity with Rv0812, respectively. Overlaying all three active sites, we noted a conserved set of core catalytic residues—K149/K145/K140, E182/E177/E173, and R50/R50/R46—in Rv0812, *Bs*DAAT, and *Pa*ADCL, respectively (Fig. 2 D); however, there were unique features of the Rv0812 active site that help explain its dual specificity. The active sites of *Bs*DAAT and *Pa*ADCL both include a long loop of substrate-interacting residues that extend from the opposing subunit (Fig. 2 B). In *Bs*DAAT, two residues in this loop—Arg98* and His100*—close to the –OH of PLP, have been proposed to comprise a carboxylate trap (Peisach et al., 1998), required for correct positioning of incoming substrates. *Pa*ADCL also contains a 17-residue long loop, structurally similar to that of *Bs*DAAT, but which is involved in stabilizing PLP-OH via a Tyr92* residue (O’Rourke et al., 2011). In contrast, Rv0812 encodes a much shorter loop that consists of only nine residues (L7: Arg95*–Pro103*), none of which appear to interact directly with substrates for either reaction (Fig. 2 B).

In light of this difference, we determined the structure of Rv0812 bound to its DAAT products in order to elucidate the structural basis of substrate binding by Rv0812. Rv0812 crystals produced after incubation with PLP and D-Glu were isomorphous with the PLP-containing crystals (Table S1) and contained electron density corresponding to pyridoxamine 5′-phosphate (PMP) and AKG in the active site, allowing the cofactor and product to be manually built in Emsley and Cowtan (2004). The PMP-AKG bound structure revealed that Arg98* in *Bs*DAAT (Figs. 2 C and S1 N), which formed H-bonds and salt bridges with the AKG C5 (2.7 Å) and D-Ala carboxylates (3.3 Å, 3.4 Å), corresponding to Arg26* in Rv0812, while Arg115 and Arg185 donated H-bonds (2.4 Å, 2.8 Å) to the AKG C5 carboxylate, and Arg115 (2.7 Å) and Thr255 (3.0 Å) stabilized the AKG C1 carboxylate (Fig. S1, J and K). These interactions serve to identify a structurally distinct, but functionally analogous, carboxylate trap in Rv0812, while allowing for potentially broader substrate specificity (Figs. 2 and S1, J and N).

Experimental efforts to obtain crystals of Rv0812 bound to PABA were unsuccessful; however, in silico modeling of Rv0812 using the structure of *Pa*ADCL enabled manual docking of ADC into the AKG binding site of the PMP-AKG bound structure. Notwithstanding known limitations of energy minimization–based docking studies (that include protein structure rigidity and the absence of coordinating water molecules), this model revealed a series of ADC-coordinating residues that notably included the carboxylate trap residues Arg26*, Arg115, and Arg185, which appear positioned to stabilize the C9 carboxylate of ADC through a network of H-bonds and salt bridges (2.8 Å, 3.3 Å, 3.1 Å), while Arg 115 (2.3 Å), Ser252 (2.7 Å), and Thr255 (2.5 Å) appear within H-bonding distance of the C5 carboxylate of ADC, and Arg26* and Thr33 appear within H-bonding distance of the oxygen next to the C1 olefin (2.3 Å) and amino group (3.1 Å) of ADC, respectively (Fig. S1 L). An overlay of the Rv0812-AKG complex structure with that of *Pa*ADCL conversely revealed a Tyr residue (Tyr22*) at the position corresponding to Arg26*, but Tyr22 was nearly 5 Å away from the AKG C5 carboxylate (Fig. S1 P).

Interestingly, ADCL enzymes from *E. coli* and *P. falciparum*, both of which contain the conserved Y22 residue, have previously been shown to act on D-Ala (Jhee et al., 2000; Magnani et al., 2013). Due to their extremely low efficiencies with D-Ala, however, these activities were proposed to be moonlighting functions derived from a shared ancestral active site. The turnover rate of D-Ala transamination by the *E. coli* ADCL was specifically found to be 700-fold lower than that of Rv0812, and catalytic efficiency of *P. falciparum* ADCL was more than 30-fold less than that of Rv0812 (Fig. 1 E). Comparison of Rv0812’s kinetic parameters with those of Y22-containing ADCL enzymes with apparent moonlighting DAAT activities thus supports a critical role for the R26* residue in mediating physiological levels of DAAT activity.

Putting the foregoing structural model to test, we generated and characterized the catalytic activity of a purified recombinant mutein of Rv0812 harboring a nonconservative substitution (R26A*) in a key residue of its putative carboxylate trap. Consistent with its predicted interaction with substrates of both its DAAT and ADCL activities, we observed reproducible reductions in the apparent turnover rates (*k_ca_*_t_) of both reactions in the R26A mutant compared with WT protein (Fig. S1 H). DAAT activity of the R26A variant exhibited a greater impact on *k*_cat_ (0.09 ± 0.2 s^-1^) over *K*_M, D-Ala_ (2 ± 1.3 mM), suggesting a mechanistic role for the carboxylate trap in formation of a productive enzyme-substrate complex (Fig. 1 E). Furthermore, the heightened impact of the R26A substitution on the DAAT activity of Rv0812, specifically, supports the notion that it is responsible for boosting DAAT activity to the extent that it is physiologically relevant.

### Bioinformatic reanalysis of Rv0812 phylogeny

**Figure 3.**
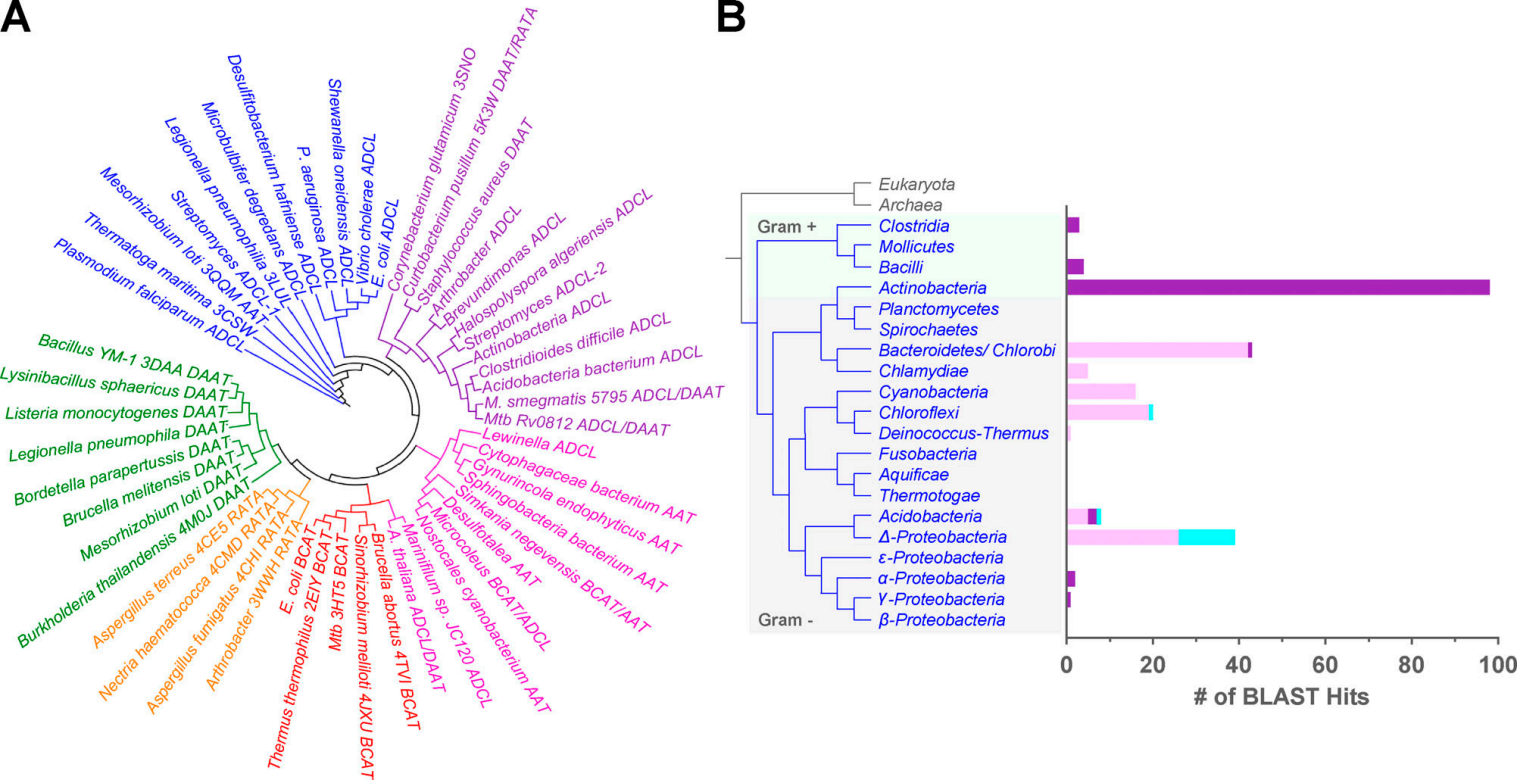
**Rv0812 characterization enables discovery of new subgroups within the type IV PLPDE family. (A)** The cladogram of type IV PLPDEs shows separation into distinct subgroups with different functions. ADCL-, DAAT-, branched chain amino acid transaminase–, and R-stereospecific amine transaminase–like sequences are colored in blue, green, red, and orange, respectively. Rv0812-like sequences were discovered by BLAST searches against each bacterial phylum shown in B and filtered to select only sequences containing Arg26. Phylogenetic analysis resulted in separation of Rv0812 orthologues into two distinct clades. Those with high similarity to Rv0812 are shown in purple (Clade I), while those with lower similarity are in pink (Clade II). National Center for Biotechnology Information annotations are provided and PDB accession nos. are shown for those with solved structures. Sequences were aligned using ClustalW2, and the tree was created using the neighbor-joining method with BLOSUM62. **(B)** The distribution and frequency of Rv0812-like orthologues across bacterial phyla was estimated by plotting the number of filtered BLAST hits (of 100 maximum) per phylum (total bar height). The number of sequences that clustered to Clade I is shown in purple, while the number of sequences in Clade II or neither group are shown in pink and cyan, respectively.

The structural identification of active site residues associated with the dual ADCL/DAAT activities of Rv0812 prompted a bioinformatic search for additional bifunctional orthologues (Marchler-Bauer et al., 2017). This resulted in the discovery of orthologous sequences containing the Arg26 residue across various bacterial phyla. A phylogenetic sequence analysis of type IV PLPDEs that included both ADCL and DAAT-like sequences further revealed that Rv0812 orthologues formed two distinct and novel clades. The clade containing Rv0812 (Clade I) clustered with a handful of annotated ADCL-like sequences but away from the majority of canonical ADCLs, while the second clade (Clade II) appeared to have more similarity to transaminase subfamilies within type IV PLPDEs (Fig. 3 A; Jhee et al., 2000; O’Rourke et al., 2011). Phylogenetic mapping across bacterial phyla further revealed that the majority of Clade I orthologues were found in gram-positive species, while Clade II orthologues were only found in gram-negative species (Fig. 3 B), suggesting the divergent evolution of a distinct, but yet-to-be determined, activity.

**Figure S2.**
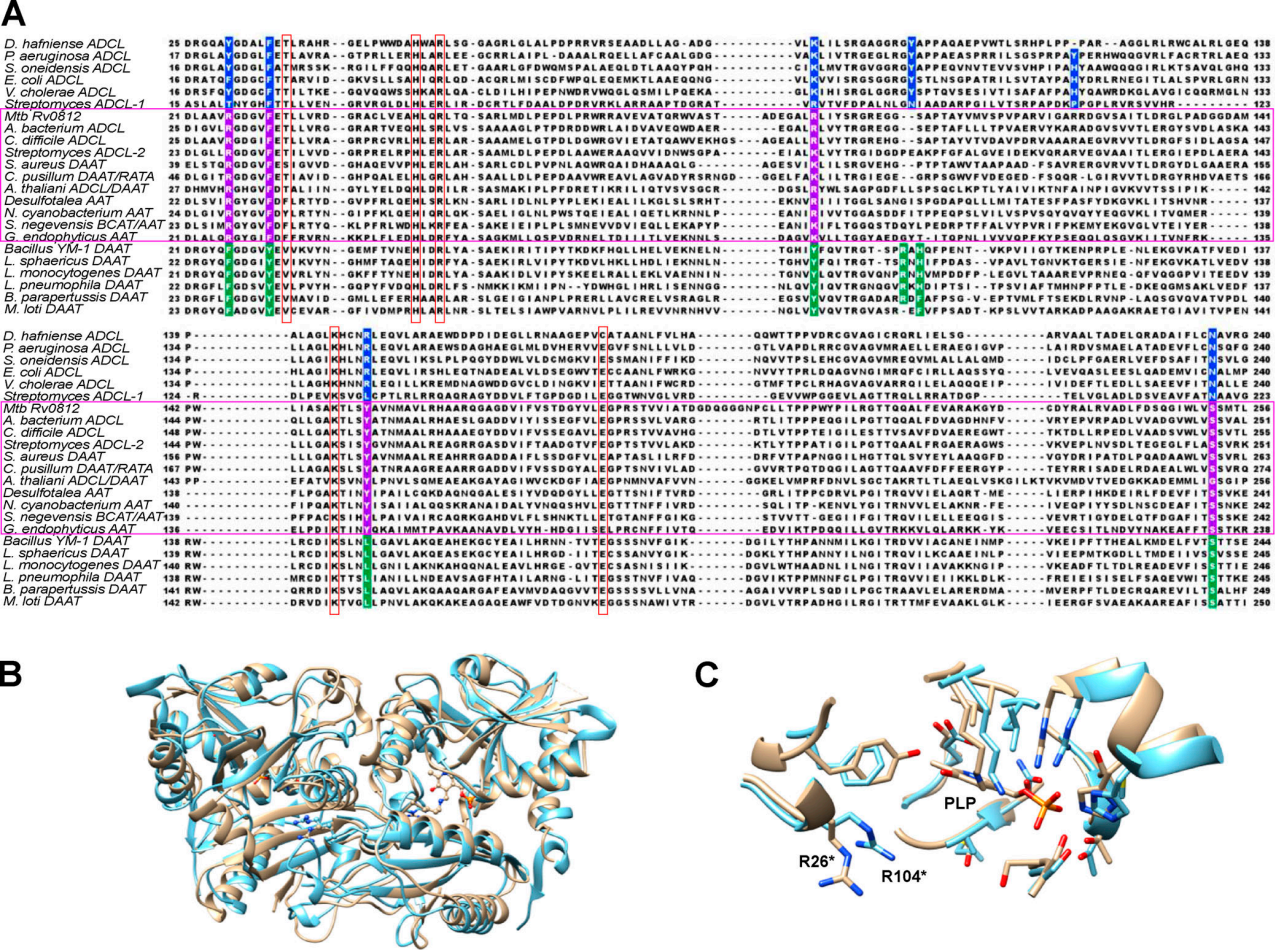
**Sequence alignment of Rv0812 with ADCL- and DAAT-like proteins from selected genomes.** **(A)** ClustalW2 multisequence alignment of *Mtb* Rv0812-like sequences compared with ADCL- and DAAT-like sequences from *D. hafniense*,* P. aeruginosa*,* S. oneidensis*,* E. coli*,* V. cholerae*,* Streptomyces*, *A. bacterium*, *C. difficile*, *S. aureus*, *C. pusillum*, *A. thaliani*, *Desulfotalea*, *N. cyanobacterium*, *S. negevensis*, *G. endophyticus*, *Bacillus YM-1*, *L. sphaericus*, *L. monocytogenes*, *L. pneumophila*, *B. parapertussis*, and *M. loti.* Sequences within the magenta box clustered along with Rv0812 in the phylogenetic tree, while known ADCL and DAAT sequences grouped with their respective subfamilies and are thus shown above and below Rv0812-like sequences, respectively. Residues conserved across all families are shown in red boxes. Conserved ADCL-like residues are shaded in blue, while DAAT-like residues are green and Rv0812-like residues are purple. **(B and C)** Superposition of Rv0812 (tan) and a homology model of *A. thaliana* ADCL/DAAT (blue). The ribbon diagram overlay, with the conserved Arg26* (Rv0812) and Arg104* (*A. thaliana* ADCL/DAAT) residues as ball and sticks, shows the structural similarity between the dually functional enzymes, with a 3.88-Å root-mean-square deviation between all atoms (B). A closer view of the residues within 4 Å of PLP demonstrates the high degree of similarity between the two active sites (C).

Previous analyses had identified two separate subclasses of ADCL-like enzymes based on the subunit contributions of the active site Tyr92/Tyr153 residue, with one subclass deriving its active site Tyr residue from the same subunit forming the active site (Position I) and the other subclass contributing the active site Tyr to the opposite protomeric subunit (Position II; O’Rourke et al., 2011). *Pa*ADCL belongs to the former subclass, while Rv0812 is a member of the latter. Surprisingly, the two ADCL clusters observed in our analysis did not conform to this classification, as members of the Position II subclass were observed in both clusters, though all sequences in the Rv0812 cluster belonged to the Position II subclass (Waterhouse et al., 2009). We found instead that all Rv0812-like, ADCL-like sequences encoded an Arg (R26*) rather than Tyr (Y22*) at the equivalent position, in addition to several other conserved sequence differences (Fig. S2). The Rv0812-containing cluster thus appears to constitute a novel group of the Position II subclass, with sequences that share greater active site similarity to DAATs.

Awaiting future studies of this nascent subclass of bifunctional ADCL-like sequences, studies of the ADCL from *Arabidopsis* *thaliana*, which encodes the same conserved active site residues as Rv0812 (Fig. S2 A), have separately reported evidence of ADCL activity in one study and DAAT activity in another (Basset et al., 2004; Funakoshi et al., 2008). Moreover, superpositioning the active site of Rv0812 with that of an in silico model of ADCL/DAAT from *A. thaliana* (Fig. S2 B) further revealed a high degree (eight of 12 residues) of conservation of residues lining the cofactor binding site, while the spatial position of the catalytically important Arg26* of Rv0812 differed from that of Arg104* in the *A. thaliana* structure by less than 1.1 Å among all nearby main chain atoms (Fig. S2 C).

It is similarly interesting to note the presence of two annotated ADCL (*pabC*)-like sequences in the *Streptomyces* genome (Zhang et al., 2009). The canonical PabC-1 sequence, encoded by a gene located near *pabAB*, shows the conservation of the canonical ADCL Y22 and N236 residues, while the PabC-2 sequence, phylogenetically clustered with Rv0812, does not. It is unknown whether PabC-1 and -2 exhibited DAAT activity, but the unusual occurrence of two ADCL sequences in the genome and differences in their active site conservation suggest an additional DAAT function for PabC-2, rather than redundancy.

### Physiological characterization of Rv0812

**Figure 4.**
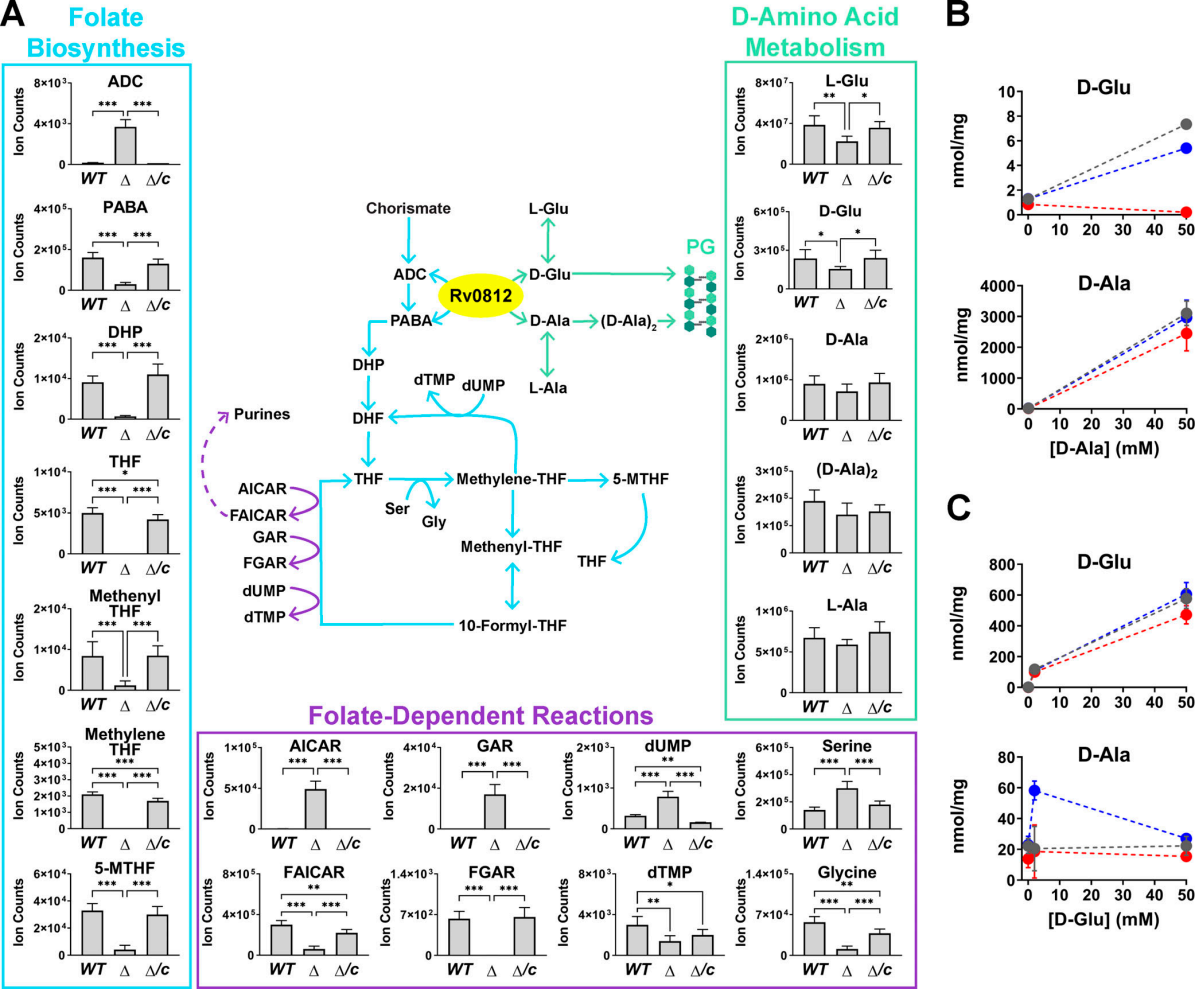
**Rv0812 impacts *Mtb* folate and D–amino acid metabolism. (A)** *Mtb ΔRv0812* is significantly impaired for folate biosynthesis and exhibits minor defects in D–amino acid metabolism. Intracellular metabolite abundance levels within *Mtb* WT, *ΔRv0812* (*Δ*), and *ΔRv0812::Rv0812* (*Δ/c*) strains grown in Sauton’s media. Whereas the *ΔRv0812* strain displayed only minor deficiencies in D–amino acid levels (green), loss of Rv0812 conferred severe defects within folate biosynthesis (blue) and downstream folate-dependent reactions (purple). Data are presented as means ± SD of triplicate samples from two separate experiments. *, P ≤ 0.05; **, P ≤ 0.01; ***, P ≤ 0.001 by one-way ANOVA and Tukey’s multiple comparisons test. **(B and C)** Rv0812 exhibits DAAT activity in vivo. Intracellular concentrations of D-Ala or D-Glu are shown in response to supplementation with 0, 2, and 50 mM D-Ala (B) or D-Glu (C). Intracellular metabolites from WT *Mtb* are shown in gray, while metabolites within *ΔRv0812* and *ΔRv0812::Rv0812* are shown in red and blue, respectively. SDs were obtained from at least two independent experiments. THF, 5, 6, 7, 8-tetrahydrofolate; DHP, dihydropteroate; 5-MTHF, 5-methyl tetrahydrofolate; AICAR, 5-Amino-1-(5-phospho-beta-D-ribosyl)imidazole-4-carboxamide; dUMP, deoxyuridine monophosphate; dTMP, deoxythymidine monophosphate; FGAR, N2-Formyl-N1-(5-phospho-D-ribosyl)glycinamide.

**Figure S3.**
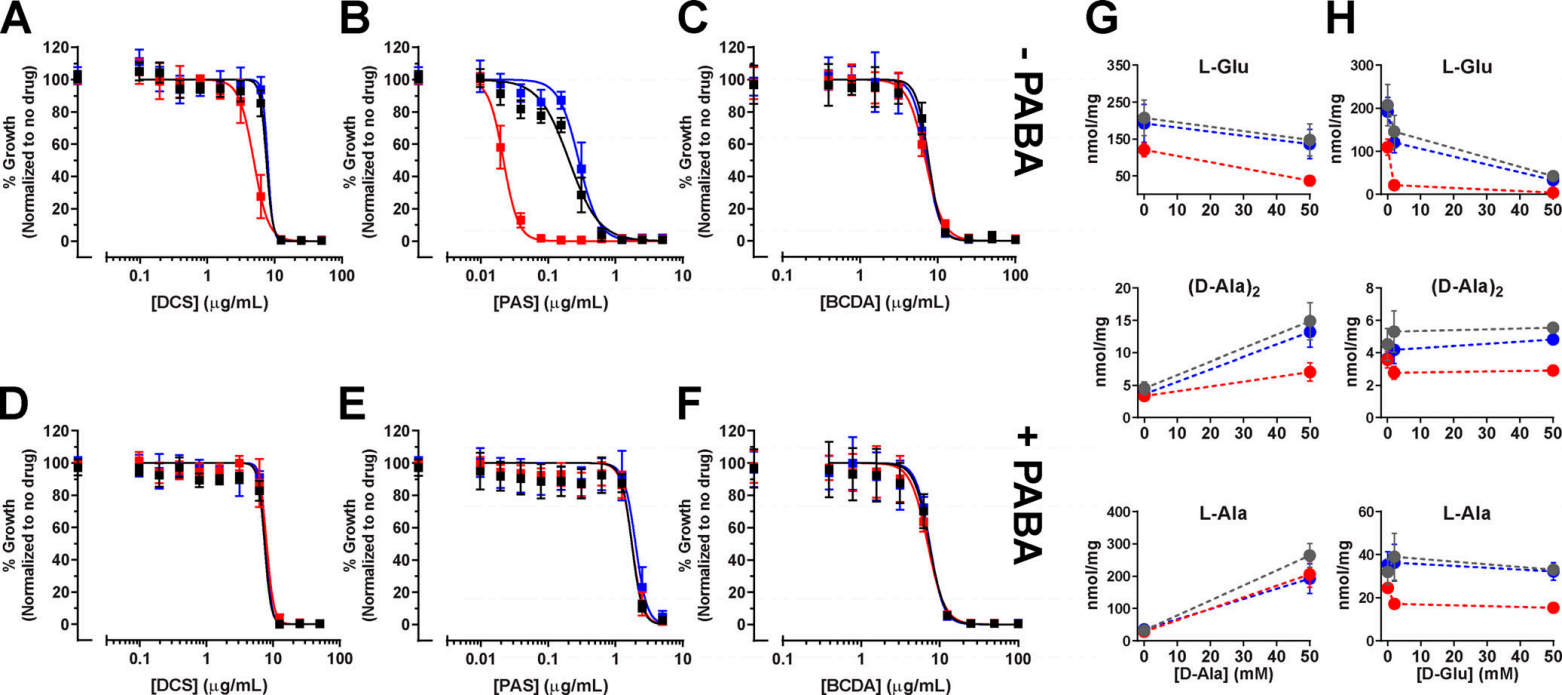
***Mtb ΔRv0812* is hypersusceptible to PAS, not DCS or BCDA. (A–F)** The minimum inhibitory concentrations of DCS (A and D), PAS (B and E), and BCDA (C and F) against *Mtb* WT (black), *ΔRv0812* (red), and *ΔRv0812::Rv0812* (blue) were determined in the absence (A–C) or presence (D–F) of PABA (1 µg/ml). Cultures grown to mid-log phase were diluted into fresh Sauton’s media to a starting OD_580 nm_ of 0.01 and OD was measured after 10 d. MIC_90_ represents the drug concentration allowing 10% of maximum growth. No significant change in MIC of DCS or BCDA was observed across strains, independent of PABA supplementation (MIC_90_ = 8.5–10 µg/ml for DCS and 15 µg/ml for BCDA). However, *Mtb ΔRv0812* was hypersusceptible to PAS (MIC_90_ = 0.03 µg/ml) compared with WT and *ΔRv0812::Rv0812* strains (MIC_90_ = 0.6 µg/ml), which was recovered by PABA supplementation (MIC_90_ = 3 µg/ml). SDs were obtained from at least two independent experiments. **(G and H)** Intracellular concentrations of metabolites from D–amino acid metabolism are shown in response to supplementation with 0, 2, and 50 mM D-Ala (G) or D-Glu (H). Intracellular metabolites from WT *Mtb* are shown in gray, while metabolites within *ΔRv0812* and *ΔRv0812::Rv0812* are shown in red and blue, respectively. SDs were obtained from at least two independent experiments.

Phylogenetic predictions notwithstanding, we sought evidence of a physiological linkage between these two biochemically distinct activities. To do so, we generated a precise isogenic deletion of Rv0812. Consistent with previous reports of an *ΔRv0812* mutant in *H37Ra* and a transposon insertion mutant in *H37Rv*, *ΔRv0812 Mtb* exhibited a growth defect in liquid culture that could be corrected by expression of an extragenic copy of the WT allele or chemical addition of PABA to the culture medium (Fig. S1 I; Thiede et al., 2016). Comparative metabolic profiling further revealed a marked and selective accumulation of ADC, while the levels of PABA and downstream intermediates of folate biosynthesis were markedly depleted compared with WT and Rv0812 reconstituted strains (Fig. 4 A). These changes were further linked to a >10-fold increase in susceptibility to the antifolate, para-aminosalicylic acid (PAS; Fig. S3 B). These results establish a nonredundant, physiological role for Rv0812 in de novo folate biosynthesis.

Seeking physiological evidence of DAAT activity in vivo, we performed metabolomic profiling of WT, *ΔRv0812*, and *ΔRv0812::Rv0812* strains incubated in the presence of either exogenous D-Ala or D-Glu. *ΔRv0812 Mtb* exhibited a selective and genetically complementable defect in D-Glu pools, but not D-Ala pools (Fig. 4 A). Incubation with exogenous D-Ala further revealed linked Rv0812-dependent increases in L-Ala, (D-Ala)_2_, and D-Glu (Figs. 4 B and S3 G), whereas reciprocal effects of D-Glu supplementation were not observed (Figs. 4 C and S3 H). These findings suggest that under the conditions tested, Rv0812 operates in the direction D-Glu synthesis. This directionality and physiological role are consistent with the nearly sixfold higher *k*_cat_ of Rv0812 for D-Ala than D-Glu as a substrate, near equivalent *k*_cat_ of *Mtb* alanine racemase (Alr) for D- and L-Ala as substrates, and general kinetic preference of bacterial *Mtb* glutamate racemase (MurI) enzymes for L-Glu over D-Glu. Moreover, where determined, D-Glu pools have been reported to be ∼10-fold lower than L-Glu pools, suggesting that D-Glu synthesis is tightly regulated at low levels, whereas L- and D-Ala pools are maintained at near equal concentrations, consistent with apparent equilibrium position—or ratio—of their *k*_cat_s. To further validate our findings, we determined what, to our knowledge, are the first measurements of the effective aqueous intrabacterial concentrations of D-Ala and D-Glu (2.1 ± 1.01 and 0.15 ± 0.05 mM, respectively) and L-Ala and L-Glu (2.9 ± 1.65 and 21 ± 10 mM, respectively) in *Mtb*. These values and near unit ratio of forward and reverse reaction rates of *Mtb*’s Alr support a basal or failsafe role for Rv0812 in coupling D-Ala and D-Glu synthesis to one another—a finding physiologically supported by the selective defect in D-Glu pools observed in Rv0812-deficient strains. In addition, the apparent lack of D-Glu–induced changes in D-Ala pools may be explained by the nearly 39-fold-higher turnover rate of D-alanyl-D-alanine ligase (Ddl) than that of Rv0812 (*k*_cat_ = 9.7 s^−1^ and *k*_cat, D-Glu_ = 0.252 s^−1^ for Ddl and Rv0812, respectively; Prosser and de Carvalho, 2013c). These results nonetheless collectively demonstrate the physiological competency of the DAAT activity of Rv0812.

**Figure 5.**
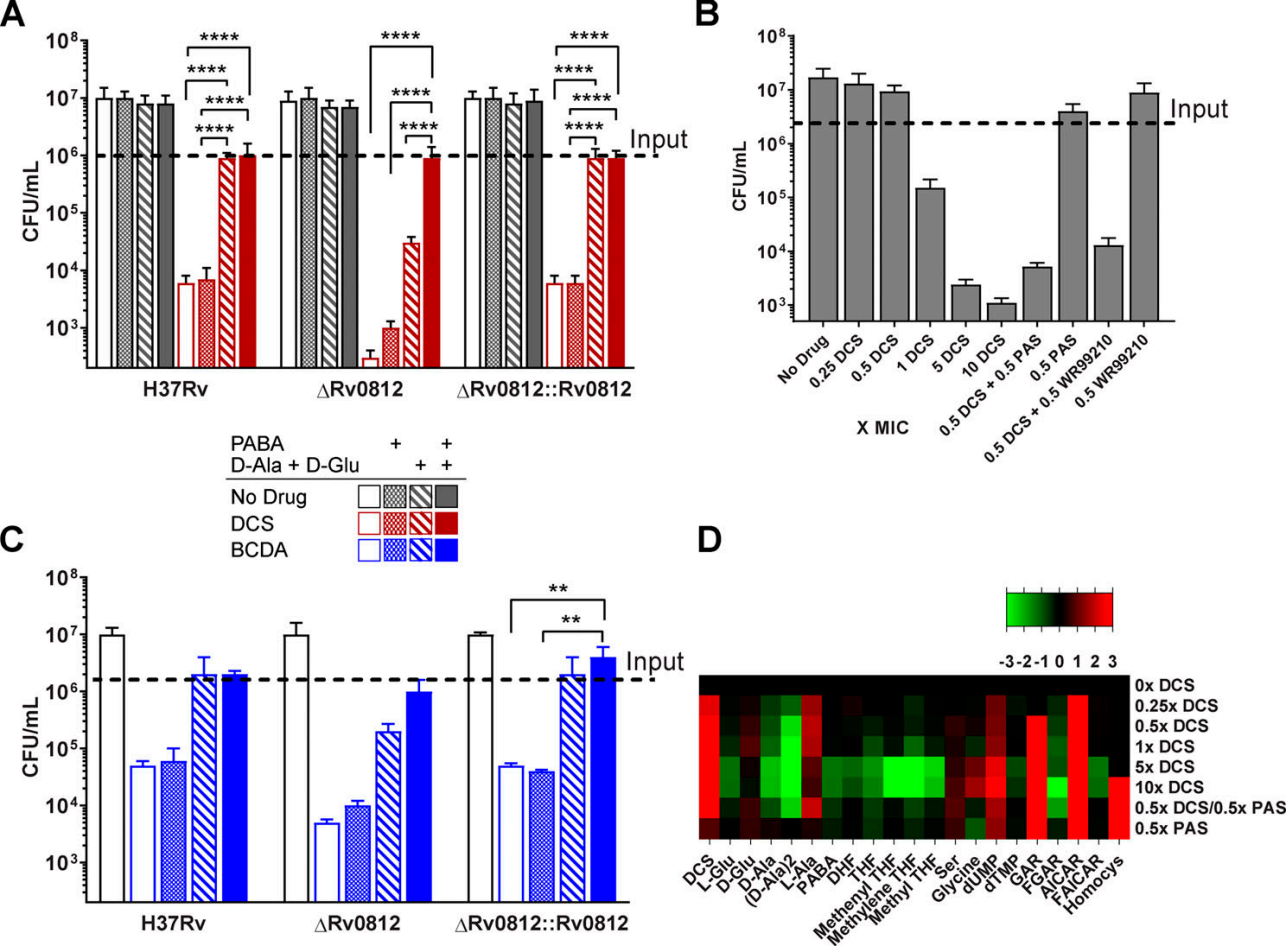
**Rv0812 bifunctionality provides a failsafe mechanism for coupling folate and PG biosynthesis. (A and C)** *Mtb ΔRv0812* hypersensitivity to PG inhibitors is rescued by supplementation with PABA, D-Ala, and D-Glu. Comparison of CFUs of *Mtb* WT, *ΔRv0812*, and *ΔRv0812::Rv0812* in the absence (gray bars) or presence of (A) DCS (red bars; 60 µg/ml) or (C) BCDA (blue bars; 120 µg/ml). Moving left to right across groups, survival in the presence of drug was compared with the extent of phenotypic rescue by supplementation with 1 µg/ml PABA, D-Ala, and D-Glu (2 mM each); or PABA, D-Ala, and D-Glu. Data are presented as means ± SD of triplicate samples from two separate experiments. **, P ≤ 0.01; ****, P ≤ 0.0001 by two-way ANOVA and Tukey’s multiple comparisons test. **(B and D)** Antifolate signature corresponds to cidality. WT *Mtb* was exposed to varying concentrations of DCS, or 0.5× MIC doses of PAS or WR99210 in the absence or presence of 0.5× MIC doses of DCS. **(B)** CFUs following 6 d of drug exposure. **(D)** Heatmap demonstrating the antifolate signature and impact on D-amino acids observed at cidal DCS concentrations. SDs were obtained from at least two independent experiments.

Given the reported positive or alleviating epistatic interactions of Rv0812 with several annotated genes of PG metabolism (DeJesus et al., 2017a; Kieser et al., 2015; Xu et al., 2017), we also sought to directly test the functional importance of the DAAT activity of Rv0812 in PG biosynthesis. To do so, we tested the susceptibility of *ΔRv0812* to D-cycloserine (DCS) and β-chloro-D-alanine (BCDA), two validated whole-cell active inhibitors of *Mtb* PG biosynthesis (David, 2001; Manning et al., 1974; Prosser and de Carvalho, 2013a). DCS is a clinically approved second-line TB drug whose mode of action is mediated by inhibition of Alr and Ddl (Prosser and de Carvalho, 2013b), while BCDA was recently shown to act as a whole cell-active, mechanism-based inhibitor of MurI (Prosser et al., 2016). Consistent with their established primary targets, we observed no difference in the minimum inhibitory concentration of either DCS or BCDA against *ΔRv0812* (Fig. S3, A and C). Following exposure to supra-MIC (minimum inhibitory concentration) levels, however, we noted that the *ΔRv0812* mutant exhibited an additional 1 log_10_ loss of viability compared with either the WT or complemented strain for each compound, indicating an essential and specific role for the DAAT activity of Rv0812 in *Mtb* viability when Alr or MurI racemase activity is absent. We further showed that this enhanced susceptibility to DCS or BCDA could be rescued, in part, by the addition of exogenous D-Ala and D-Glu, or PABA, to the culture medium (Fig. 5, A and C). These results provide further physiological evidence of Rv0812’s activity as a bidirectional DAAT.

Restoration of WT levels of susceptibility to either DCS or BCDA in *ΔRv0812 Mtb* required the joint addition of exogenous D-Ala, D-Glu, and PABA (Fig. 5, A and C). Given the chemically and mechanistically distinct nature of DCS and BCDA and the fact that PABA alone did not cause any measurable degree of rescue from DCS or BCDA in WT *Mtb*, this requirement suggested that the increased susceptibility of *ΔRv0812 Mtb* to both DCS and BCDA was due to a loss of both the DAAT and ADCL activities of Rv0812.

Interestingly, DCS exhibited a larger impact on *ΔRv0812 Mtb* than BCDA. Recent work has shown that the antimycobacterial activity of DCS is mediated through the inhibition of multiple targets (de Chiara et al., 2020). We profiled the metabolomic impact of DCS on WT *Mtb* during the prelethal phase of treatment and discovered a specific impact on intermediates and downstream products of folate metabolism at bactericidal, but not bacteriostatic, concentrations (Fig. 5 D). We further observed that treatment of WT *Mtb* with subinhibitory concentrations of DCS combined with subinhibitory concentrations of either PAS or WR99210 (a whole-cell inhibitor of *Mtb*’s dihydrofolate reductase; Nixon et al., 2014) resulted in a >5 log_10_ reduction in *Mtb* viability (Fig. 5 B). This synergy suggests that the increased susceptibility of *ΔRv0812 Mtb* to DCS than BCDA is due to its additional inhibition of *Mtb* folate biosynthesis. Moreover, this increased susceptibility reveals a previously unrecognized biological coupling of PG and folate biosynthesis in *Mtb* that the enzymatic bifunctionality of Rv0812 appears poised to serve as an enzymatic failsafe defense.

### Potential implications of a bifunctional ADCL/DAAT

Owing to their essentiality in bacteria and absence in humans, folate and PG biosynthesis pathways have served as highly validated antitubercular drug targets for decades (Gautam et al., 2011; Green and Matthews, 2007). Knowledge of specific physiological links between these two target pathways, however, has remained unaddressed. Like all cells, bacteria face the challenge of needing to coordinate growth and division to ensure successful replication. For *Mtb*, this challenge is complicated by the erratic, albeit repetitive, nature of its cell cycle, in which replication often follows a prolonged, unpredictable interval of host-imposed nutritional deprivation and biochemical stress (Ehrt et al., 2018; Smith, 2003).

Our discovery of Rv0812 as a bifunctional ADCL and DAAT reveals a previously unrecognized metabolic coupling between nucleic acid and cell wall biosynthesis that appears to ensure prioritization of PABA production over D-Ala/D-Glu biosynthesis. This prioritization is evidenced by the phenotypic auxotrophy of *ΔRv0812* for PABA rather than D-Ala or D-Glu. Moreover, because both ADCL and DAAT activities are catalyzed by the same active site chemistry (Percudani and Peracchi, 2009), Rv0812 appears enzymatically poised to function as a metabolic toggle that alternates between ADCL and DAAT activity, prioritizing the former over the latter in response to substrate accumulation. From a structural perspective, this bifunctionality appears to have been selected for, in part, by the loss of conserved residues Y22, Y/H112, and N236 specific to ADCLs (Nakai et al., 2000; O’Rourke et al., 2011; Parsons et al., 2002). The reaction rates of its DAAT activity additionally indicate that Rv0812 kinetically favors the production of D-Glu five times more than D-Ala. This preference is further supported by the greater degree of Rv0812-dependent D-Glu accumulation in *Mtb* supplemented with D-Ala than that of D-Ala and (D-Ala)_2_ in culture medium containing D-Glu. As all transaminase reactions are thermodynamically freely reversible, the equivalent vulnerability of *ΔRv0812* to inhibition of either D-Ala or D-Glu biosynthesis, combined with its strong affinity for D-Glu and the previously reported synergy between DCS and BCDA against WT *Mtb*, indicate that Rv0812 has evolved to ensure balanced production of both D-Ala and D-Glu (David, 2001). Moreover, the demonstrated ability of Rv0812 to catalyze the interconversion of D-Ala and D-Glu in vivo identifies a previously unrecognized role for its DAAT activity in defense against PG-targeting drugs.

The competition between ADCL and DAAT substrates for the active site of Rv0812 further reveals a new physiological link between nucleic acid and PG biosynthesis. This link is manifested by the increased vulnerability to DCS- or BCDA-mediated killing and the shared requirement for both PABA and D-Ala/D-Glu to restore WT levels of susceptibility, despite acting through a distinct set of molecular targets. This link is further reinforced by the marked, multi-log synergy of DCS and two mechanistically distinct antifolates when combined at subinhibitory concentrations against WT *Mtb*, as well as the heightened susceptibility of *ΔRv0812 Mtb* to the dual impact of DCS on D-Ala and folate biosynthesis (Chakraborty et al., 2013; Nixon et al., 2014).

While powerful, an underappreciated limitation of homology-based gene annotations is their inherent bias toward evolutionarily invariant functions and limited ability to reveal functions related to more phylogenetically specific selective pressures. This is because conservation is an end product, rather than a driving force, of evolution. Yet organisms evolve their genomes in response to the specific selective pressures they encounter. Existing bioinformatic methods have therefore not sufficed to reveal the most distinguishing physiological features of a given organism. For pathogenic microbes, such as *Mtb*, such features correspond to a biologically ideal but untapped source of potential diagnostic biomarkers and drug targets. The discovery of ADCL-like enzymes with homology to Rv0812 across a wider range of bacteria suggests that coordination of bacterial growth and division may be a more broadly conserved, but previously unannotated, metabolic activity.

## Materials and methods

### Strain construction

The *rv0812* gene deletion mutant was constructed by allelic exchange via homologous recombination, as previously described (Gee et al., 2012), replacing the native copy of *rv0812* with a zeocin resistance cassette. Mutant candidates were isolated from Middlebrook 7H10 solid culture medium supplemented with 1 µg/ml PABA and confirmed via Southern blot analysis. The *rv0812* complemented strain was constructed by reintroducing a copy of *rv0812* under the control of the *hsp60* promoter into the attL5 site of the *Mtb* genome. *Mtb* WT (H37Rv) and mutant strains were routinely grown in Sauton’s medium with 0.04% tyloxapol, and, when necessary, zeocin and kanamycin were added to cultures at final concentrations of 25 µg/ml and 20 µg/ml, respectively.

### Protein expression and purification

The sequence of the full-length Rv0812 gene was amplified from the *Mtb* H37Rv genome by PCR. The amplified gene was inserted into a pMCSG19B expression vector containing an N-terminal 6x-His tag using ligation-independent cloning sites (Eschenfeldt et al., 2009). The Rv0812:R26A and Rv0812:Y153A mutants were constructed by amplifying pMCSG19B-Rv0812 plasmids using the QuickChange II XL Site-Directed Mutagenesis Kit (Agilent Technologies). Plasmids were transformed into BL21(DE3) *E. coli* cells for protein expression. Cells with plasmids were grown at 37°C to an OD_600_ of ∼0.6–0.8 in Difco LB medium (Becton Dickinson) with 100 µg/ml carbenicillin followed by induction with 500 µM isopropyl β-D-1-thiogalactopyranoside and grown overnight at 18°C.

The sequence of the full-length *Ec*PabB gene was amplified from a BL21(DE3) single colony by PCR. The amplified gene was inserted into the same plasmid as Rv0812 using the same method as described. The plasmids were transformed into BL21(DE3) *E. coli* cells for *Ec*PabB expression. The cells with the plasmid were grown at 37°C to an OD_600_ of ∼0.6–0.8 in Difco LB medium with 100 µg/ml carbenicillin followed by induction with 1 mM isopropyl β-D-1-thiogalactopyranoside and grown for 6.5 h at 37°C.

Cells were lysed via a Microfluidizer M-100P (Microfluidics) in lysis buffer (50 mM Tris [pH 7.5], 500 mM NaCl, 10% glycerol, 25 mM imidazole, 2 mM 2-mercaptoethanol, 1 mM PMSF, 10 µg/ml DNase, and 2 mM MgCl_2_) and centrifuged at 27,216 ×*g* for 1 h. The supernatant was purified over a nickel column with a 0–500-mM imidazole gradient, followed by size-exclusion chromatography using elution buffer (25 mM Tris [pH 7.5], 100 mM NaCl, 10% glycerol, 2 mM dithiothreitol). The proteins were >95% pure, as observed by SDS-PAGE, and were concentrated to 5 mg/ml, flash frozen, and stored in elution buffer at −80°C. Protein concentrations were determined by measuring A_280_ using a NanoDrop 2000 UV-visible spectrophotometer (Thermo Fisher Scientific) using extinction coefficients of 37,930 M^−1^cm^−1^ and 56,755 M^−1^cm^−1^ for *Mtb* Rv0812 and *Ec*PabB, respectively.

### Activity-based metabolomic profiling (ABMP)

*Mycobacterium bovis* Bacillus Calmette-Guerin was grown in 7H9 medium supplemented with 0.2% glycerol, 0.5% BSA, 0.2% dextrose, and 0.085% NaCl. A 9-liter culture grown to OD_580 nm_ of 1.0 was harvested by centrifugation, and the resulting pellet was resuspended in 100 ml of an acetonitrile (ACN):methanol:H_2_O (40:40:20) solution. Cells were lysed using an Emulsiflex C5 high-pressure homogenizer (Avestin) and centrifuged for 20 min at 20,000 ×*g*. Soluble extract was lyophilized and resuspended in 10 ml 25 mM Tris-HCl (pH 7.4), yielding the final small-molecule extract (SME).

For ABMP analysis, 150-µl reactions containing 75 µl SME and 5 µM purified recombinant Rv0812 (active or heat killed for 15 min at 95°C) were incubated in 100 mM Tris-HCl (pH 8.5) at 37°C in the presence of 50 µM PLP and 1 mM D-Ala or D-Glu. At indicated time points, reaction aliquots were quenched with cold ACN containing 0.2% formic acid for a final concentration of 80% quenching solution. After centrifugation at 20,000 ×*g* for 10 min, the resulting supernatant was separated from insoluble material and stored at 4°C for liquid chromatography-mass spectrometry (LC-MS) analysis.

### Mass spectrometry

For separation and detection of metabolites, LC-MS analysis was conducted using an Agilent 1200 LC system containing a Cogent Diamond Hydride Type C silica column (150 mm × 2.1 mm; Microsolv Technologies) coupled to an Agilent Accurate Mass 6220 TOF as described (Eoh and Rhee, 2013). The mobile phase consisted of solvent A (double-distilled H_2_O with 0.2% formic acid) and solvent B (ACN with 0.2% formic acid) at a flow rate of 0.4 ml/min with the following gradient: 0–2 min, 85% B; 3–5 min, 80% B; 6–7 min, 75% B; 8–9 min, 70% B; 10–11.1 min, 50% B; 11.1–14 min, 20% B; and 14.1–24 min, 5% B; followed by a 10-min equilibration period at 85% solvent B before injection of the next sample. Dynamic mass axis calibration was accomplished by continuous infusion of a reference mass solution. Electrospray ionization capillary and fragmentor voltages were set at 3,500 V and 135 V, respectively. The nebulizer pressure was set to 40 psig and nitrogen drying gas was maintained at 250°C, set to a flow rate of 10 liter/min. The MS acquisition rate was 1.5 spectra/s and m/z data ranging from 50 to 1,700 was stored. Data were analyzed using Profinder B.08.00 software, and ions were assigned as specific metabolites based on mass accuracy within 5 ppm and retention times within 1 min of those determined for chemical standards.

### Enzymatic activity assays

Kinetic measurements of Rv0812’s transaminase activity were obtained by means of two distinct assays, according to the substrate being consumed in the reaction. An LC-MS–based assay was used for kinetic analysis using D-Glu as the substrate. Reactions (200 µl) contained 5 mM MgCl_2_, 5 mM pyruvate, 50 µM PLP, 0.25 µM WT-Rv0812, and varying concentrations of D-Glu (50 µM to 10 mM) in 100 mM Tris (pH 8.5). At indicated time points, reaction aliquots were quenched with cold ACN containing 0.2% formic acid for a final concentration of 80% quenching solution. After centrifugation at 20,000 ×*g* for 10 min, the resulting supernatant was separated from insoluble material and stored at 4°C for LC-MS analysis.

The kinetic parameters of Rv0812’s transaminase activity, using D-Ala as a substrate, were determined by using an LDH-coupled assay. Reactions (200 µl) were performed at 25°C in 100 mM Tris (pH 8.5) containing 5 mM MgCl_2_, 5 mM AKG, 5 U/ml LDH, 1 mM nicotinamide adenine dinucleotide (NADH), 50 µM PLP, and 0.25 µM WT-Rv0812 or mutants. The buffer was premixed and incubated for 30 min. The assay was initiated by adding 25 µl D-Ala for final concentrations ranging from 31 µM to 32 mM. Enzyme activity was monitored by the decrease in absorbance at 340 nm, representing NADH consumption, using a Varioskan LUX multimode microplate reader (Thermo Fisher Scientific).

Kinetic measurements of Rv0812’s ADCL activity were likewise obtained by using an LDH-coupled assay. ADCL activity assays were performed at 25°C in 100 mM Tris (pH 8.5) containing 5 mM MgCl_2_, 100 mM (NH_4_)_2_SO_4_, 5 U/ml LDH, 500 µM NADH, 25 µM PLP, 11 µM *Ec*PabB, and chorismate at concentrations ranging from 0 to 2 mM. The buffer was premixed and incubated for 30 min. The assay was initiated by adding 0.5 µM WT-Rv0812 or mutants. PABA production was monitored using a Varioskan LUX multimode microplate reader (Thermo Fisher Scientific) at 340 nm to monitor the decrease in NADH concentration. For quantitative analysis of pyruvate production, only the 2 mM chorismate group was used.

For in vitro DAAT and ADCL kinetics assays, experiments were performed in triplicate for each protein construct and reported as average ± SEM. Kinetic data were fitted with the Michaelis-Menten equation (Michaelis et al., 2011) using the JMP Pro 13 Software (SAS Institute).

### Substrate competition assays

Substrate competition assays were conducted as described above in the presence or absence of varying concentrations of substrates. ADCL assays were conducted in the presence of *Ec*PabB and 0.4 mM or 1.6 mM chorismate as a surrogate for the ADC substrate in the presence or absence of D-Ala (0, 7.5, or 30 mM) or D-Glu (0, 0.25, or 1 mM) as competing substrate. DAAT assays were conducted in the presence of D-Ala (7.5 or 30 mM) or D-Glu (0.25, or 1 mM) in the presence or absence of *Ec*PabB and chorismate (0, 0.4, or 1.6 mM) as competing substrate. Kinetic parameters in the presence or absence of competing substrate were determined by plotting the inverse velocity against the inverse substrate concentration on double reciprocal plots and calculating the inverse of the x- and y-intercepts.

### Phylogenetic analysis

BLAST searches using the Rv0812 amino acid sequence were conducted against various bacterial phyla and classes with a maximum output of 100 hits for each group. BLAST hits were screened to include only those sequences with conservation of Arg26 as potentially Rv0812 like. Sequences were aligned with representative members of each type IV PLPDE subfamily using Clustal W2 and were subsequently placed into a phylogenetic tree using the neighbor joining method with BLOSUM62 to predict functional annotations. Phylogenetic trees were visualized using the interactive tree of life program (Letunic and Bork, 2019).

### In vivo metabolomic profiling of *Mtb* strains

In vivo metabolic profiling of *Mtb* strains was conducted as previously described (Eoh and Rhee, 2013). A 1-ml culture (OD_580 nm_ = 1.0) of each strain was collected on a nitrocellulose filter and grown on 7H10 plates for 5 d at 37°C. Filters were subsequently transferred to swimming pools containing ∼3 ml of Sauton’s medium in the presence or absence of PABA (1 µg/ml), DCS, PAS, WR99210, D-Ala, and/or D-Glu. Filters were collected following 24 h of exposure, and metabolic activity was quenched by placing cells in 1 ml of an ACN:methanol:H_2_O (40:40:20) solution. Cells were lysed by mechanical disruption with 0.1-mm Zirconia beads (BioSpec Products) in a Precellys tissue homogenizer (cooled to 4°C) for 3 min at 6,500 rpm three times. Centrifugation enabled the separation of soluble cellular metabolites, which were then filtered through Spin-X (0.22 µM) columns for removal from the BSL3 facility. Metabolite extracts were diluted 1:1 in LC-MS solvent B, centrifuged for 10 min at 10,000 ×*g*, and supernatant (2 µl) was injected onto a Diamond Hydride column for LC-MS analysis as described above. For normalization of ion abundance to cell biomass, the residual protein concentration in lysates was quantified (BCA Protein Assay Kit; Thermo Fisher Scientific).

### Drug susceptibility assays

*Mtb* cultures grown to mid-log phase in Sauton’s medium were diluted in fresh medium to a starting OD_580 nm_ of 0.01. Varying concentrations of compounds in DMSO were added at 1% of the total volume. Following 6 d of exposure to compounds, cultures were serially diluted with 1× PBS containing 0.04% tyloxapol. Serial dilutions were streaked on 7H10 plates and allowed to recover for 21 d, at which point colonies were counted at appropriate dilutions.

### Protein crystallization

Rv0812 was first crystallized without incubation with cofactor or compounds. Crystallization conditions (formulated by Hampton Research) were screened using a Mosquito liquid dispenser (TTP Labtech) using the sitting drop vapor diffusion technique in the Crystal Mation Intelli-Plate 96–3 low-profile crystallization plate (Hampton Research). For each condition, 0.4 µl protein (5 mg/ml) and 0.4 µl crystallization formulation were mixed, and the mixture was equilibrated with 50 µl crystallization solution in the reservoir well. Full-length WT Rv0812 protein crystals were further optimized via hanging drop vapor diffusion by incubating 2 µl purified protein solution (5 mg/ml) with 1 µl crystallization solution (1 M sodium citrate and 0.1 M sodium cacodylate [pH 7.1]) at 18°C for 3 d. Crystals formed as clusters during the first few optimizations. The microseeding method was applied to acquire larger and well-separated single crystals (Luft and DeTitta, 1999). A crystal cluster was taken from the optimized condition along with 10 µl mother liquor. The cluster was then crushed by vortexing for 3 min with a Seed Bead kit (Hampton research). The mixture was added to 90 µl mother liquor to prepare the 1:10 seed stock, which was further diluted to make 1:100 and 1:1,000 seed stocks. New crystals were obtained via hanging drop vapor diffusion by incubating 2 µl purified protein solution (3 mg/ml) with 1 µl seed stocks. The mixture was equilibrated with optimized crystallization conditions (1 M sodium citrate and 0.1 M sodium cacodylate [pH 7.1]) at 18°C for 3 d. The best crystals appeared in 1:1,000 dilution seed stock. These crystals were cryo-protected with 23% ethylene glycol and flash frozen before data collection. For PLP complex crystals, 0.2 mM PLP (pH adjusted to 6.6) was cocrystallized with 3 mg/ml Rv0812 using the above procedure. For PMP and AKG-bound crystals, 1 mM D-Glu and 0.2 mM PLP (pH adjusted to 6.6) were cocrystallized with 3 mg/ml Rv0812 using the same method as for PLP-bound crystals.

### Data collection and structure determination

Data were collected at Argonne National Laboratory using the Advanced Photon Source beamlines 19ID and 23ID-D. All data were processed and reduced using HKL2000 (Otwinowski and Minor, 1997). The structure of apo-Rv0812 was solved by molecular replacement using MOLREP (Vagin and Teplyakov, 2010) in CCP4 (Winn et al., 2011) using the coordinates for the fold IV-transaminase (CpuTA1) from *Curtobacterium pusillum* from the PDB (accession no. 5K3W; Pavkov-Keller et al., 2016). The PLP-bound and PMP and AKG-bound structures were solved by molecular replacement using MOLREP in CCP4 with the solved apo-Rv0812 structure. Refinement and manual model building were performed with PHENIX (Adams et al., 2010) and COOT (Emsley and Cowtan, 2004), respectively.

### Molecular docking

The simplified molecular-input line-entry system (SMILES) string of ADC was created by the JSME Structure Editor (Bienfait and Ertl, 2013) and the coordinates were generated through the CADD Group SMILES Translator (Sitzmann et al., 2008). ADC was placed into the active site of the PMP-containing Rv0812 structure—AKG, waters, and other ligands were removed from the model before docking—in COOT (Emsley and Cowtan, 2004). ADC was incorporated into the location according to the bound PMP. The active site docking model with the lowest global energy minimum was generated by the MolSoft ICM Chemist Pro Docking Program (Orry and Abagyan, 2012).

A model of the *A. thaliana* ADCL/DAAT was generated from the amino acid sequence of the enzyme using the Phyre2 webserver (Kelley et al., 2015). Structural superposition of Rv0812 with *A. thaliana* ADCL/DAAT was performed using Chimera (Pettersen et al., 2004).

### Macromolecular assembly analysis

After acquiring Rv0812 structures, both apo- and PLP-bound structure coordinate files were uploaded to the program PDBePISA (Krissinel and Henrick, 2007) to analyze the macromolecular assemblies. Interactions across the interfaces were found. The buried surface area of the interfaces and the free energy of assembly dissociation (ΔG^diss^) were calculated for both structures.

### Data and software availability

The Apo, PLP-bound, and PMP and AKG-bound structures were deposited in the PDB (Berman et al., 2000) with accession nos. 6Q1Q, 6Q1R, and 6Q1S, respectively.

### Online supplemental material

Fig. S1 provides additional evidence for the ability of Rv0812 to serve as both a DAAT and ADCL in the form of kinetic, structural, and phenotypic analysis. Bioinformatic analysis and structural homology modeling shown in Fig. S2 suggest that Rv0812 and similar sequences constitute a novel subgroup of type IV PLPDEs that are defined by their proposed ADCL/DAAT bifunctionality. Fig. S3 provides additional evidence for the ability of Rv0812 to serve as an ADCL and DAAT in vivo. Table S1 lists detailed parameters of the crystallography data and refinement statistic.

## Supplementary Material

Table S1 lists detailed parameters of the crystallography data and refinement statistic.
