# Supplementary material for: Metabolic bifunctionality of Rv0812 couples folate and peptidoglycan biosynthesis in Mycobacterium tuberculosis
Source: J Exp Med. 2021 May 5;218(7):e20191957. doi: 10.1084/jem.20191957 (PMC8105722; doi:10.1084/jem.20191957)
Supplement: Table S1 — lists detailed parameters of the crystallography data and refinement statistic. [file JEM_20191957_TableS1.docx]

|  | **Apo-Rv0812** | **PLP-Rv0812** | **AKG-PMP-Rv0812** |
| --- | --- | --- | --- |
| **Data collection** |  |  |  |
| Source | APS, 23ID-D | APS, 19ID | APS, 23ID-D |
| space group | P2_1_ | P2_1_ | P2_1_ |
| cell dimensions |  |  |  |
| a, b, c (Å) | 48.4, 66.5, 97.5 | 66.4, 203.2, 48.1 | 66.5, 202.3, 48.1 |
| α, β, γ (deg) | 90, 90.1, 90 | 90, 90.0, 90 | 90, 90.0, 90 |
| Resolution | 48.8-2.4 (2.48-2.40) | 40.3-2.7 (2.80-2.70) | 48.1-2.3 (2.40-2.30) |
| R_merge_ (%) | 13.3 (45) | 40.7 (68) | 13.9 (64) |
| I/σ(I) | 9.75 (2.67) | 5.32 (1.14) | 6.59 (1.54) |
| completeness (%) | 99.51 (97.14) | 98.84 (97.69) | 99.86 (99.93) |
| **Refinement** |  |  |  |
| no. of molecule in ASU | 2 | 4 | 4 |
| no. of reflections | 24428 | 33555 | 56131 |
| Rwork/Rfree | 0.19/0.23 | 0.22/0.26 | 0.20/0.23 |
| no. atoms | 4525 | 8765 | 9041 |
| ligand | 2 | 6 | 9 |
| water | 157 | 81 | 306 |
| B-factors | 48.62 | 50.47 | 25.41 |
| rms deviations |  |  |  |
| bond lengths (Å) | 0.003 | 0.004 | 0.002 |
| bond angles (deg) | 0.67 | 0.77 | 0.61 |
| Ramachandran favored (%) | 98.93 | 98.12 | 98.12 |
| Ramachandran allowed (%) | 1.07 | 1.88 | 1.52 |
| Ramachandran outliers (%) | 0 | 0 | 0.36 |

**Table SI. Crystallography Data and Refinement Statistics**
